# Can Limited Education of Lung Ultrasound Be Conducted to Medical Students Properly? A Pilot Study

**DOI:** 10.1155/2017/8147075

**Published:** 2017-03-28

**Authors:** Jang Sun Lim, Sanghun Lee, Han Ho Do, Kyu Ho Oh

**Affiliations:** Department of Emergency Medicine, Dongguk University College of Medicine, Dongguk University Ilsan Hospital, Goyang-si 10326, Republic of Korea

## Abstract

*Objectives*. Lung ultrasonography (LUS) is a useful examination to identify lung problems. Unfortunately, there are currently no LUS educational programs for medical students. We designed a brief LUS training course for medical students during the ED rotation. The purpose of training was improving cognitive and psychomotor learning domains, knowledge of ultrasound, knowledge of LUS, image acquisition, and image interpretation.* Methods*. Forty students in their fourth year of medical school were enrolled in this study. Student achievement was evaluated through examinations of cognitive and psychomotor skills. A survey was administered following the training.* Results.* The average test result was 42.1 ± 13.7 before training and 82.6 ± 10.7 after training. With respect to the assessment of LUS performance, the acceptable rates for right and left anterior chest wall scanning and right and left posterolateral scanning were 95%, 97.5%, 92.5%, and 100%, respectively. The students felt a high level of confidence in their ability to administer LUS to patients after training and they agreed that inclusion of LUS training in the medical school curriculum is necessary.* Conclusion.* This study showed that, among the medical students without ultrasound experience, limited LUS education to improve their knowledge, image acquisition, and interpretation ability was successful.

## 1. Introduction

The use of ultrasonography by clinicians is increasing because it is convenient, fast, and noninvasive and aids in decision making and the performance of other procedures [1–4]. Among the many point of care ultrasound (POCUS) examinations, lung ultrasonography (LUS) is the most useful for detecting lung problems at the bedside [5–7]. Using LUS, a variety of diseases such as pneumothorax, pulmonary edema, pleural effusion, and pneumonia can be diagnosed rapidly. LUS is shown to be useful not only in POCUS examination to uncover etiology of respiratory failure but also in monitoring hemodynamics and alveolar recruitment and guiding pleural procedures [8–11]. In spite of this advantage, LUS is not included in most medical school curriculums.

We designed a brief LUS training course for medical students during the ED rotation. The purpose of training was improving cognitive and psychomotor learning domains, knowledge of ultrasound, knowledge of LUS, image acquisition, and image interpretation.

## 2. Materials and Methods

### 2.1. Study Design

This prospective study was performed from April to June of 2015 at one medical college in South Korea. The subjects in this study were 40 students who were undergoing clerkship at the emergency department (ED) during their fourth year in medical school. The students were divided into five groups of eight students each, and training was conducted for each group. Written consent was obtained from students, and approval for this study was obtained from the Institutional Review Board (IRB) of author's hospital.

The authors developed the education program and the assessment methods to evaluate the effectiveness of training based on current recommendations of LUS and BLUE protocol described by Lichtenstein et al. [5, 12]. Through numerous meetings and revision over a period of one month, the information gathered through this progress was used to develop the contents, the evaluation guidelines, and posttraining survey.

In order to evaluate the effectiveness of this program, the authors gave the students a written test on knowledge and interpretation ability before and after the training, a hands-on LUS performance test, and surveys after the completion of training.

### 2.2. Validity

Content, face, and construct validity were checked.

Content validity was assessed by surveying six members of local lung ultrasonography experts (3 critical care medicine physicians and 3 emergency physicians). They were asked to rate the appropriateness of each item based on a 4-point Likert scale (1 = not relevant, 2 = somewhat relevant, 3 = quite relevant, and 4 = highly relevant). Then, for each item, the content validity index (CVI) was computed as the number of experts giving a rating of either 3 or 4; CVI of 0.83 or higher was selected.

Eight emergency medicine (EM) residents of varying grade were selected for assessing face validity. They completed pretest, posttest, and posttraining survey; then their comments and points of view were recorded. Based on their comments and feedbacks from the experts, minor editorial changes were made to increase the clarity of the items.

Evidence of construct validity was obtained by applying the posttest to a convenience sample of eight EM residents of varying grade.

### 2.3. Training for Lung Ultrasonography

Training was performed by an emergency physician, an ultrasonography education director at the ED of the authors' hospital who had 10 years of experience in emergency ultrasonography and POCUS education. The educational course lasted for a total of 3 hours, with 1 hour of training on LUS theory and 2 hours of hands-on training.

In the didactic tutorial, the basic principles and operational methods necessary for a LUS examination were explained, and the methods and interpretation of ten normal and abnormal sonographic findings of the lung which were described by Lichtenstein. Normal and abnormal sonographic findings include the bat sign (pleural line), lung sliding (yielding seashore sign), the A-line (horizontal artifact), the quad sign, and sinusoid sign indicating pleural effusion, the fractal, and tissue-like sign indicating lung consolidation, the B-line, and lung rockets indicating interstitial syndrome, abolished lung sliding with the stratosphere sign suggesting pneumothorax, and the lung point indicating pneumothorax [5].

Hands-on training was performed by applying a transducer to a healthy individual, not a patient. The LUS examination was performed on the right side of the subject while he was in a supine position. LUS scan was consistent with anterior examination and posterolateral examination similar to the exam described in the Bedside Lung Ultrasound in Emergency (BLUE) protocol, scan of the upper and the lower BLUE point and the posterolateral alveolar and/or pleural syndrome (PLAPS) point. The sequence of the anterior examination was as follows. (1) Upper anterior point: a transducer was placed at the intersection of the mid-clavicular line and the second intercostal space. (2) Lower anterior point: a transducer was placed on the anterior axillary line at approximately the fifth intercostal space. Scanning of both anterior examination points was performed longitudinally to the thorax, perpendicular to the pleura. When inspecting the scans, two cross-sections of the ribs were shown on the right and left sides of the screen, and the pleura was observed in a downward position between the ribs. This association of ribs and pleural line make a land mark called the bat sign, which was the basic image used for lung ultrasonography. Then examiner observed the lung sliding sign (the movement of pleura was observed during respiration) and A-lines (horizontal artifacts indicating a normal lung surface that arise from the reverberation between the pleura and the transducer). The following procedure was used for the posterolateral examination: a transducer was placed at the intersection of the connecting line with both lower anterior examination point and the posterior axillary line, and scanning was performed while holding the transducer in a longitudinal direction, aimed anteriorly. As in the anterior scan, examiner checked lung sliding sign and A-lines on the pleura between two ribs.

The obtained images were stored as videos 3 seconds in duration. SONOACE X8® (Samsung Medison, Co., Ltd., Korea) and a curvilinear 2–8 MHz (C2-8, Samsung Medison, Co., Ltd., Korea) probe were used.

### 2.4. Evaluating the Effect of Education

A pretraining test consisting of 40 questions was administered, and a posttraining test with a similar composition was conducted to assess any changes in knowledge. The 40 questions were based on LUS learning goals. There were 6 questions on theory and physics, 3 on knobology, 11 on normal lung anatomy, and 20 on pathological findings of pneumothorax, pulmonary edema, pneumonia, and pleural effusion. A total of 31 questions concerning normal anatomy and pathological findings were given using images and videos.

The ability to perform lung ultrasonography was evaluated by considering students' competence regarding the skills performed and the accuracy of the images obtained by the checklist (see Supplementary Material available online at https://doi.org/10.1155/2017/8147075). Anterior examination and posterolateral examination in both lungs were classified as success, failure, or acceptable. When the performance on the LUS examination and the obtained images were appropriate, the examination was judged to be successful. If there were some insufficiencies in the instrument operation or image acquisition, but the images were clinically readable, the test was judged to be acceptable. If there were deficits in the performance and image acquisition, or if the images were not obtained or were impossible to read, the examination was judged to be a failure. Competence about the skills performed was evaluated by a fourth-year emergency medical resident during a hands-on test and the properties of the images obtained were judged by an ultrasound education director at the ED. After completing LUS training, students were given a survey to determine their satisfaction, understanding of the training course, opinions about the need for LUS education during medical school, and confidence in their ability to perform LUS on patients. The questions were rated on a 5-point Likert scale, with the possible answers being “strongly disagree (1 point), disagree (2 points), neutral (3 points), agree (4 points), and strongly agree (5 points).”

### 2.5. Statistical Analysis

Categorical variables are presented as frequency and percentage. The normality of continuous variables was confirmed by the Kolmogorov-Smirnov test. Continuous variables are expressed as averages and standard deviations in cases in which data was normally distributed, and they are indicated as medians and quartiles if data had an irregular distribution. A paired* t*-test was performed to confirm the improvement in knowledge level of the students before and after training. Independent* t*-test was performed to obtain construct validity. Statistical analysis was performed using the SPSS version 18 (IBM Inc., Chicago, USA) program, and a* p* value less than 0.05 was considered statistically significant.

## 3. Results

### 3.1. General Characteristics

A total of 40 students received LUS training. All of them completed the written pretest and posttest, performance test of LUS, and a posttraining survey. The average age of the students was 27.4 ± 3.1 years, and they consisted of 24 males and 16 females. Although all students had received education in ultrasonography previously, none of them had experience with hands-on training. None of the students reported that they had any previous knowledge about lung ultrasonography (Table 1).

### 3.2. Cognitive Skills: Knowledge of Ultrasound, Knowledge of LUS, and Image Interpretation

**Figure 1:**
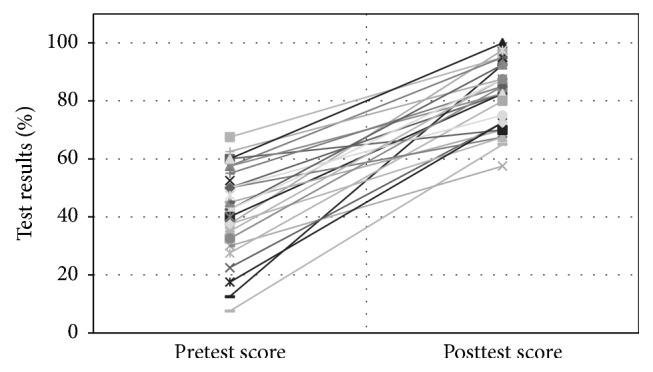
Comparison of pretest and posttest scores. Improvement in the medical students' posteducational knowledge status about the physiology of an ultrasound examination and their ability to interpret LUS findings. The mean score on the pretest was 42.1 and the mean score on the posttest was 82.6.

The average pretraining test result was 42.1 ± 13.7 and the average posttraining test result was 82.6 ± 10.7, indicating a significant improvement in knowledge regarding sonography after education (*p* < 0.001) (Figure 1).

### 3.3. Construct Validity for Knowledge of Ultrasound and LUS

There were no statistically significant differences between students' and residents' overall posttest scores (82.6 ± 10.7 versus 88.4 ± 15.1, *p* = 0.33).

### 3.4. Psychomotor Skills: Image Acquisition

**Figure 2:**
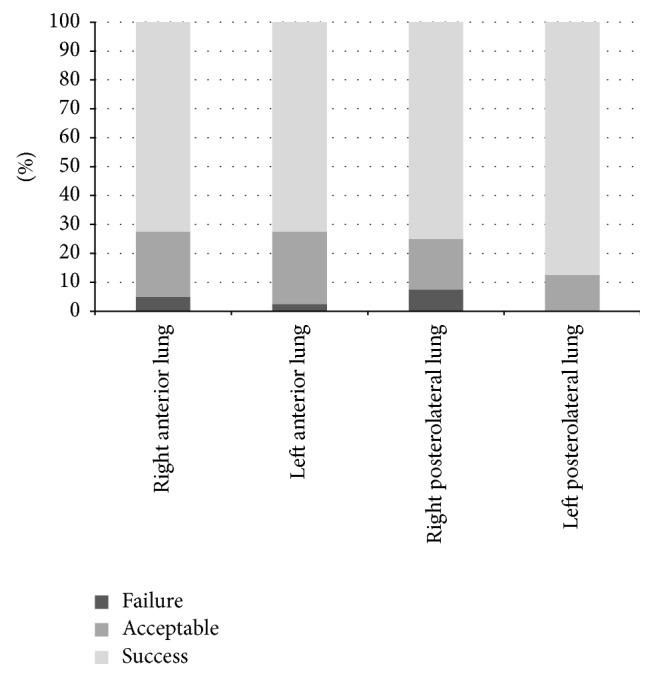
Success rate of the LUS hands-on session. The rates of acceptable or better performance of right and left anterior chest scanning and right and left posterolateral scanning were 95% and 97.5% and 92.5% and 100%, respectively.

With respect to the assessment of LUS performance ability, the acceptable rates for right anterior chest wall scanning, left anterior chest wall scanning, and right and left posterolateral scanning were 95%, 97.5%, 92.5%, and 100%, respectively (Figure 2). The survey completed after LUS education revealed that the students felt that the training was easy and the programs were satisfactory.

### 3.5. Student Responses: Posttraining Survey

The students felt a high level of confidence in their ability to administer LUS to patients after training and they agreed that inclusion of LUS training in the medical school curriculum is necessary (Table 2).

## 4. Discussion

In this study, after a short-term limited LUS training, we observed that novice medical students were able to perform LUS to normal healthy volunteer and to interpret LUS images properly. Sonographic examination of the lungs can provide instant clues that allow physicians to differentiate between pneumothorax, pleural effusion, pneumonia, and pulmonary edema. As such, it is valuable when treating acute-stage dyspneic patients [12–14]. However, thus far, no study has assessed the effect of LUS training given to medical school students.

Beaulieu et al. [15] provided theory education for 2.5 hours and hands-on training for 2 hours to junior emergency medicine residents, and they reported a significant effect of training on the ability to perform LUS. We obtained similar results from our LUS training program for medical students. The students in this study showed major improvements in their knowledge of ultrasound principles and ability to interpret the results of LUS. The assessment scores before and after education were 42.1 ± 13.7 and 82.6 ± 10.7, respectively, which demonstrate significant improvement. These results were similar but the improvement was greater than those in previous reports on Focused Assessment with Sonography for Trauma (FAST) education provided to novice medical students by Arger et al. [16] and Bentley et al. [17]

The success rate of LUS performance among students was very high and there were no differences according to examination position. The success rate of LUS in this study was 96.3%, which was higher than the success of FAST education found by Gogalniceanu et al. [18] who reported that 25 medical college students showed a success rate of 86.0%. Blackstock et al. [19] also reported 89% pass rates for FAST skill assessment with forty-five medical students on an EM rotation. We believe that the higher success rate of LUS is due to sonographic access to superficially located pleura being relatively easy and transducer handling being simpler than that necessary for the FAST examination.

The POCUS examinations performed by clinicians have recently increased; hence, studies assessing the feasibility of ultrasound education in medical school have been carried out [16, 20–25]. This study first showed that LUS knowledge and scanning skill was effectively imparted to students. It was also identified through a survey given after training that students understood the training course easily and they were satisfied with the training. The students reported that they had high confidence in their ability to perform LUS on real patients after training. And they reported that inclusion of lung ultrasonography training in their medical school curriculum is necessary. There are many who argue that ultrasound training should be introduced into medical education curriculum [26–31]. The authors believe that LUS training should be one of the first line candidates if undergraduate ultrasonography education is established. This study can be cornerstone when the medical school curriculum about POCUS is developed.

This study has some limitations. First, it is difficult to generalize the results because the sample size was small, consisting of only 40 students, and the study included students from only a single medical college. Second, the students could not observe pathologic lesions directly when performing ultrasonography. The purpose of the performance assessment was to visualize normal anatomical structures in a healthy volunteer, it was not possible to evaluate whether students would be able to scan and interpret pathologic findings in real patients. Third, we did not evaluate retained knowledge after a period of time, because our research was conducted only in clerkship period at the emergency department (ED). Future studies will need to be supplemented by evaluation after a certain period of time. The final limitation was that the students performed LUS in healthy volunteer. This may have made it easier for students to obtain images than it would be in real clinical settings.

## 5. Conclusion

We report on the successful limited LUS education to improve medical students' knowledge, image acquisition, and interpretation ability. These results can support that medical students are able to learn limited LUS with brief exposure.

## Supplementary Material

The checklist was applied to student to evaluate the performance of LUS.

## Figures and Tables

**Table 1:** Baseline characteristics of the medical students.

Characteristics	Value
Age (years)	27.4 ± 3.1
Gender	
Male	24 (60%)
Female	16 (40%)
Previous knowledge of LUS	
Yes	0 (0%)
No	40 (100%)

**Table 2:** Student responses to the posttraining survey on LUS education.

	Likert scale^*∗*^				
	1	2	3	4	5
Understanding of LUS^†^ training	0 (0%)	4 (10%)	5 (12.5)	22 (55%)	9 (22.5%)
Satisfaction with LUS training	0 (0%)	0 (0%)	3 (7.5%)	21 (52.5%)	16 (40%)
Need for LUS training within the medical curriculum	0 (0%)	0 (0%)	6 (15%)	20 (50%)	14 (35%)
Confidence of ability to perform LUS to patients	0 (0%)	0 (0%)	5 (12.5)	26 (65%)	9 (22.5%)

^*∗*^Likert scale;  ^†^LUS: lung ultrasound

1: strongly disagree, 2: disagree, 3: neutral, 4: agree, and 5: strongly agree.
