# Supplementary material for: Can Limited Education of Lung Ultrasound Be Conducted to Medical Students Properly? A Pilot Study
Source: Biomed Res Int. 2017 Mar 28;2017:8147075. doi: 10.1155/2017/8147075 (PMC5387807; doi:10.1155/2017/8147075)
Supplement: Supplementary file 1 — The checklist was applied to student to evaluate the performance of LUS. [file 8147075.f1.docx]

**Supplement**

| Lung ultrasound examination performance test | | Right | | | Left | | |
| --- | --- | --- | --- | --- | --- | --- | --- |
| Anterior lung examination | | S | A | F | S | A | F |
| 1 | **Proper probe location**  -Success: a transducer was placed at adequate position.  : upper anterior - intersection of mid-clavicular line with the 2nd intercostal space  : lower anterior - anterior axillary line at approximately 5th intercostal space  -Acceptable: did not meet the criteria, but in discrimination had no trouble.  -Fail: did not meet the criteria and could not get the proper image of the anterior side of the lung |  |  |  |  |  |  |
| 2 | **Proper probe direction**  -Success: direction of the transducer was placed by the cephalad and longitudinally to the thorax.  -Acceptable : longitudinal direction but not cephalad (caudal direction)  -Fail: transversely to the thorax. |  |  |  |  |  |  |
| 3 | **Basic image of anterior lung examination**  -Success: 2 cross-sections of the bony part of the ribs were shown on the right and  left sides of the screen, and the pleura was observed in a downward position  between the ribs; the pleura was also visible on the screen horizontally.  -Acceptable: the chondral part of the ribs was observed, but otherwise met the  criteria. The pleura was not visible on the screen horizontally, but there was no  trouble distinguishing it.  -Fail : could not get the proper images of the anterior lung |  |  |  |  |  |  |
| 4 | **Proper images of lung sliding**  -Success : the movement of pleura was observed during respiration  -Fail: lung sliding was not observed. |  |  |  |  |  |  |
| 5 | **Proper images of A-lines**  -Success: A-lines were observed.  -Fail: A-lines were not observed. |  |  |  |  |  |  |
| Posterolateral lung examination | | S | A | F | S | A | F |
| 1 | **Proper probe location**  -Success: a transducer was placed at the point of intersection of the connecting line  with both nipples and the posterior axillary line  -Acceptable: did not meet the criteria, but there was no trouble distinguishing it.  -Fail: did not meet the criteria and could not get the proper image of the  posterolateral side of lung |  |  |  |  |  |  |
| 2 | **Proper probe direction**  -Success: the direction of the transducer was placed by the cephalad and  longitudinally to the thorax, aiming to the ceiling.  -Acceptable: longitudinal direction but not cephalad (caudal direction)  -Fail: transversely to the thorax. |  |  |  |  |  |  |
| 3 | **Basic image of the posterolateral lung exam**  -Success: 2 cross-sections of the bony part of the ribs were shown on the right and  left sides of the screen, and the pleura was observed in a downward position  between the ribs; the pleura was also visible on the screen horizontally.  -Acceptable: the chondral part of ribs was observed, but otherwise met the criteria.,  the pleura was not visible on the screen horizontally, but there was no trouble  distinguishing it.  -Fail: could not get the proper images of the posterolateral lung |  |  |  |  |  |  |
| 4 | **Proper images of lung sliding**  -Success : the movement of pleura was observed during respiration  -Fail : lung sliding was not observed. |  |  |  |  |  |  |
| 5 | **Proper images of A-lines**  -Success: A-lines were observed.  -Fail: A-lines were not observed. |  |  |  |  |  |  |

S: Success, A: Acceptable, F: Fail
